# Microglial activation in the lateral amygdala promotes anxiety‐like behaviors in mice with chronic moderate noise exposure

**DOI:** 10.1111/cns.14674

**Published:** 2024-03-11

**Authors:** Xiaoqi Peng, Yunfeng Mao, Yehao Liu, Qian Dai, Yingju Tai, Bin Luo, Yue Liang, Ruirui Guan, Wenjie Zhou, Lin Chen, Zhi Zhang, Guoming Shen, Haitao Wang

**Affiliations:** ^1^ Department of Anesthesiology, The First Affiliated Hospital of USTC, Hefei National Laboratory for Physical Sciences at the Microscale, Division of Life Sciences and Medicine University of Science and Technology of China Hefei China; ^2^ School of Integrated Chinese and Western Medicine Anhui University of Chinese Medicine Hefei China; ^3^ Auditory Research Laboratory, Department of Neurobiology and Biophysics, Division of Life Sciences and Medicine University of Science and Technology of China Hefei China; ^4^ Department of Psychiatry The First Affiliated Hospital of USTC Hefei China; ^5^ Department of Otolaryngology The First Affiliated Hospital of USTC Hefei China; ^6^ Songjiang Research Institute Shanghai Jiao Tong University School of Medicine Shanghai China

**Keywords:** anxiety‐like behaviors, chronic moderate noise exposure, electrophysiology, lateral amygdala, microglia

## Abstract

**Background:**

Long‐term non‐traumatic noise exposure, such as heavy traffic noise, can elicit emotional disorders in humans. However, the underlying neural substrate is still poorly understood.

**Methods:**

We exposed mice to moderate white noise for 28 days to induce anxiety‐like behaviors, measured by open‐field, elevated plus maze, and light–dark box tests. In vivo multi‐electrode recordings in awake mice were used to examine neuronal activity. Chemogenetics were used to silence specific brain regions. Viral tracing, immunofluorescence, and confocal imaging were applied to define the neural circuit and characterize the morphology of microglia.

**Results:**

Exposure to moderate noise for 28 days at an 85‐dB sound pressure level resulted in anxiety‐like behaviors in open‐field, elevated plus maze, and light–dark box tests. Viral tracing revealed that fibers projecting from the auditory cortex and auditory thalamus terminate in the lateral amygdala (LA). A noise‐induced increase in spontaneous firing rates of the LA and blockade of noise‐evoked anxiety‐like behaviors by chemogenetic inhibition of LA glutamatergic neurons together confirmed that the LA plays a critical role in noise‐induced anxiety. Noise‐exposed animals were more vulnerable to anxiety induced by acute noise stressors than control mice. In addition to these behavioral abnormalities, ionized calcium‐binding adaptor molecule 1 (Iba‐1)‐positive microglia in the LA underwent corresponding morphological modifications, including reduced process length and branching and increased soma size following noise exposure. Treatment with minocycline to suppress microglia inhibited noise‐associated changes in microglial morphology, neuronal electrophysiological activity, and behavioral changes. Furthermore, microglia‐mediated synaptic phagocytosis favored inhibitory synapses, which can cause an imbalance between excitation and inhibition, leading to anxiety‐like behaviors.

**Conclusions:**

Our study identifies LA microglial activation as a critical mediator of noise‐induced anxiety‐like behaviors, leading to neuronal and behavioral changes through selective synapse phagocytosis. Our results highlight the pivotal but previously unrecognized roles of LA microglia in chronic moderate noise‐induced behavioral changes.

## Graphical abstract

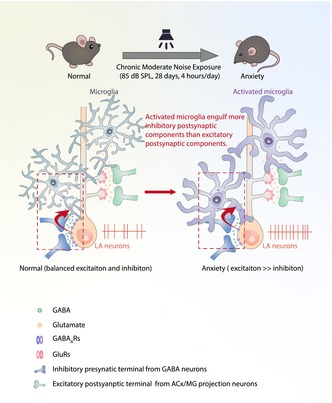

## 1 INTRODUCTION

As an unwanted sound, noise can elicit a variety of health problems, including hearing‐related and mood disorders in people.^1^, ^2^, ^3^ Short‐term exposure to high‐intensity sound of greater than 100‐decibel sound pressure level (dB SPL) often leads to hearing loss^4^, ^5^ and anxiety.^6^, ^7^ In past decades, significant attention has been paid to the mechanism underlying loud noise‐induced hearing problems. Traumatic noise can shift the tuning curve, reorganize cortical representation map, and promote synchrony of central auditory systems, contributing to the development of the tinnitus,^8^, ^9^ as well as change the hippocampus leading to the development of anxiety‐like behaviors.^7^ However, moderate noise with an intensity of <90 dB SPL is commonly encountered in traffic, nightclubs, or certain workplaces.^1^, ^10^, ^11^, ^12^ Increasing evidence supports that prolonged exposure to lower levels of noise may seem ostensibly non‐traumatic but can in fact compromise auditory processing,^13^ disturb sleep^6^ and reduce immune response,^14^ and impair learning.^15^

Of note, long‐term exposure to moderate noise has also been shown to cause emotional disorders,^16^, ^17^, ^18^ but the neural mechanism underlying these changes is not fully understood. Among the numerous limbic brain regions, the amygdala is likely the most important emotional center^19^, ^20^ and has been implicated in multiple emotional disorders, and artificial manipulation of amygdala‐related neural circuits has been shown to significantly impact emotion‐related behaviors.^21^, ^22^, ^23^, ^24^, ^25^, ^26^ Despite extensive research on the reciprocal connection between auditory brain areas and the amygdala in fear conditioning,^20^, ^27^, ^28^, ^29^ the role of the amygdala in mediating noise‐related anxiety has not been thoroughly defined. Indeed, some studies have shown that acoustic trauma increases the number of c‐Fos‐positive cells in the limbic system^30^ and that chronic stress can elevate neuronal activity in the amygdala,^31^, ^32^ thus implicating the amygdala in the induction or maintenance of noise‐related anxiety or depression.^23^, ^31^, ^33^ However, it remains unclear how chronic moderate noise exposure modulates the amygdala neural circuit.

Microglia, resident immune cells in the brain parenchyma, can respond rapidly to changes in neuronal activity to regulate synaptic connectivity through ramified processes.^34^, ^35^ Microglia dynamically mediate synaptic development and plasticity through synapse induction, synapse elimination, synaptic plasticity, clearance of extracellular matrix, and neurogenesis.^36^ Exposure to lipopolysaccharide reportedly leads to microglial activation and production of pro‐inflammatory cytokines in the basolateral amygdala (BLA), which in turn causes anxiety and depressive behaviors via synaptic and non‐synaptic plasticity.^37^ Optogenetic activation of microglia in the prefrontal cortex, amygdala,^38^ and spinal cord^39^ can trigger grooming, anxiety, and chronic pain in mice, respectively, while genetic knockout of microglial signaling molecules or microglial depletion could reverse these behavioral changes. Similarly, reactive microglia in the BLA contribute to synaptic impairment and depression‐like behavior in mice with bone cancer pain.^40^ In addition, inhibiting microglial activation in the amygdala can reverse stress‐induced abdominal pain.^41^ In noise‐related pathologies, acoustic trauma can activate microglia in the auditory stations and limbic systems,^33^ and hearing loss induced by extremely high decibel noise is associated with elevated activity in the auditory cortex mediated by increased levels of microglia‐secreted pro‐inflammatory tumor necrosis factor‐alpha.^5^ In addition, sound‐induced gamma waves in the auditory cortex and hippocampus can activate microglia to engulf plaques in a mouse model of Alzheimer's disease,^42^ and significant microglial activation was also observed in the auditory cortex and medial geniculate body of mice with salicylate‐induced tinnitus.^43^ These collective reports link microglial activation in the amygdala with noise‐related emotional disorders.

In this study, we developed a mouse model of chronic moderate noise‐induced anxiety by exposing mice to white noise at 85 dB SPL for 4 h daily for 28 days, which has been previously used to explore the non‐auditory effects of moderate noise exposure.^44^, ^45^ According to the 5‐dB exchange rate set by the Occupational Safety and Health Administration (OSHA),^46^ a 4‐h exposure at 85 dBA (A‐weighted decibels) is as dangerous as 8 h at 80 dBA, below the 90 dBA of the permissible exposure limit in the occupational noise exposure and is a typical noise level of urban traffic.^47^ We then examined microglia‐mediated synaptic remodeling in the LA of chronic moderate noise exposure mice. Multi‐electrode electrophysiological recordings revealed that the LA exhibits significant neuronal plasticity. Chemogenetic manipulation demonstrated that LA plays an essential role in anxiety‐related behaviors in this model. Immunofluorescence staining and morphological analyses showed that behavioral alterations are closely associated with microglia‐mediated synaptic phagocytosis. We propose that microglia‐mediated synaptic remodeling in the LA could underlie chronic moderate noise‐induced anxiety, expanding our understanding of environmental hazards of noise exposure.

## 2 MATERIALS AND METHODS

### 2.1 Animals

C57BL/6J and *CaMKII‐Cre* mice aged 8 to 10 weeks were purchased from Charles River or Jackson Laboratories. Because female mice have estrous cycles that can introduce variability due to hormone fluctuation, only male mice were used in the current study. Mice were housed in a colony of no more than five mice per cage in a stable environment (23–25°C ambient temperature) with free access to standard lab mouse pellet food and water on a 12‐h light/dark cycle (lights on from 07:00 to 19:00). All experimental protocols were approved by Animal Care Committee of the University of Science and Technology of China (USTC).

### 2.2 Chronic noise exposure

Mice were randomly assigned to noise exposure and control groups. The noise was created in Adobe Audition 3.2 (Adobe, USA), amplified (RX‐V359, YAMAHA, Japan), and played back over a free‐field speaker (CP‐75A, Shanghai Chuangmu). The speaker was installed above the mice cages to provide daily 4 h of 85 dB SPL white noise per day. Mouse cages were covered in mesh to ensure even distribution of the noise exposure. Noise was measured in decibels (dB) using a sound level meter (AWA‐5661‐A, Aihua, Hangzhou). The mice in the chronic moderate noise exposure group were placed in cage in a sound‐proof chamber with adequate food and water. After noise exposure, mice were returned to their normal housing room, while control mice were subjected to identical manipulations in the sound‐proof chamber but without noise stimulation.

### 2.3 Stereotaxic surgery and virus injection

Stereotaxic brain injection was conducted on the mice anesthetized by an intraperitoneal injection of pentobarbital (20 mg/kg) and mounted on a stereotactic frame (RWD, Shenzhen, China). The animals' body temperatures were maintained at 36°C throughout the surgery and virus injection with the aid of a heating pad. A small craniotomy was drilled above the target brain region based on mouse brain atlas coordinates, and a volume of 100–250 nL of the virus was delivered into the target areas at a rate of 30 nL/min through a glass micropipette with a tip size of 10–15 μm in diameter connected to a 10 μL Hamilton microliter syringe, which is controlled by a microinjection syringe pump (UMP3T‐1, WPI, USA). At the end of the injection, the pipette was rested at the injection site for an additional 5 min before withdrawal to avoid backflow of the virus. The mice's eyes were applied with the ointment for moisture throughout the experiment. The coordinates were defined as dorsoventral (DV) from the brain surface, anterior–posterior (AP) from bregma, and mediolateral (ML) from the midline (in mm).

For anterograde tracing, rAAV‐hSyn‐DIO‐mGFP‐T2A‐Synaptophysin‐mRuby‐WPRE‐hGH pA (AAV‐DIO‐mGFP‐Synaptophysin‐mRuby, 4.7 × 10^12^ viral genome (vg) mL^−1^, 200 nL, BrainVTA) or rAAV‐DIO‐mCherry‐WPRE‐hGH‐pA (AAV‐DIO‐mCherry, AAV2/9, 2.3 × 10^12^ vg mL^−1^, BrainVTA) was injected into the ACx (AP, −2.70 mm; ML, −4.90 mm with a 12°angle; DV, −0.10 mm) or the MGB (AP, −3.40 mm; ML, −2.25 mm; DV, −2.85 mm) of *CaMKII‐Cre* mice. The mice were then returned to their home cages to allow for viral expression. After 3 weeks, the mice were sacrificed, and the brains were removed for cryosection to visualize the mRuby signals from ACx^Glu^ neurons or MGB^Glu^ neurons in the LA.

To selectively silence the LA glutamatergic neurons, inhibitory chemogenetic virus of rAAV‐CaMKIIα‐hM4D(Gi)‐mCherry‐WPRE‐pA (AAV‐CaMKIIα–hM4Di–mCherry, AAV2/9, 5.85 × 10^12^ vg/mL, 180 nL) virus bilaterally injected into the LA. The rAAV‐CaMKIIα‐mCherry‐WPRE‐hGH‐pA (AAV‐CaMKIIα–mCherry, AAV2/9, 2.48 × 10^12^ vg/mL) virus was used as the control. Unless otherwise stated, all viruses were packaged by BrainVTA (Wuhan, China). All animals were transcardially perfused with ice‐cold 0.9% saline followed by ice‐cold phosphate buffer (0.1 M), including 4% paraformaldehyde (PFA). A confocal microscope (LSM880, Zeiss) captured images with fluorescence signals. Animals with missed injections were excluded from further analysis.

### 2.4 Chemogenetic manipulations

In chemogenetic experiments requiring systemic clozapine N‐oxide (CNO) administration, mice with AAV‐CaMKIIα–hM4Di‐mCherry or AAV‐CaMKIIα‐mCherry were anesthetized with isoflurane, then intraperitoneally injected with CNO (5 mg/kg, Sigma) or saline at 30 min before behavioral testing. Mice were killed after each set of behavioral tests for histological confirmation of the virus injection site. Data from mice with incorrect injection sites were excluded from further analysis.

### 2.5 Local drug infusion

A catheter (250 μm in diameter, RWD, China) was chronically implanted at brain areas of interest, such as the LA (AP, −1.85 mm; ML, 3.25 mm; DV, 3.20 mm), in anesthetized mice mounted on a stereotaxic apparatus. The implant was firmly cemented to the mouse skull and capped until drug application. An internal stainless‐steel injector was connected to the guide cannula with a diameter of 340 μm for local infusion of minocycline (100 nL, 10 mg/mL, Cat. No. M9511, Sigma‐Aldrich) or saline into the LA at a flow rate of 150 nL/min using a 10 μL syringe (Hamilton, USA) and an infusion pump. The injector was gently withdrawn 2 min after infusion, and behavioral tests were conducted 30 min later,^48^, ^49^ based on the previously reported observations that about 20 min are needed for adequate drug diffusion,^50^, ^51^ and the dose is sufficient to ensure the inhibition window for local minocycline infusion encompasses the full duration of daily noise exposure. After completing all behavioral tests, the site for the implanted catheter was also histologically confirmed. Data from mice with incorrect placement were excluded from further analysis.

### 2.6 In vivo multi‐electrode electrophysiology

In order to understand noise‐induced change of the LA neuronal activity, extracellular electrophysiology recordings were made in awake head‐fixed mice using silicon electrodes, as previously described.^45^ The mice were mounted in the stereotaxic frame under isoflurane anesthesia, and a homemade headpost was cemented to the skull. For head fixation, the headpost was firmly fastened to a holder. The mice were trained to get used to the fixation apparatus and to run freely on a plexiglass circular plate (20 cm in diameter). The mouse was anesthetized with isoflurane 1 day before the initial electrophysiological recordings, and a craniotomy was performed above the LA, which was then sealed with KWIK‐SIL silicone glue (WPI, USA) until the recording tests. On the day of the recordings, the adhesive was removed from the head‐fixed mice to expose the craniotomy, and a single‐axis micromanipulator (S‐IVM‐1500P, Scientifica, UK) remotely controlled and lowered a 16‐channel silicon probe (two shanks spacing in 200 μm, electrode site spacing in 100 μm, Jiangsu Boen medical technology, China) into the LA. Before recording, the electrodes are allowed to rest for at least 20 min. A Neurostudio amplifier and Neurostudio data acquisition software (Great Think Medical Technology, China) were used to amplify and store spike signals. Spike sorting was done using the T‐Dis E‐M algorithm integrated into Plexon's Offline Sorter 4 (USA). Neuroexplorer 5 (Nex Technologies, USA) was used to calculate the firing rates of sorted units.

### 2.7 In vitro electrophysiological recordings

Acute brain slice preparation followed the protocol described before.^52^ Mice were anesthetized entirely with pentobarbital sodium (2% w/v, i.p.) and then intracardially perfused with a 20 mL ice‐cold carbogenated modified N‐methyl‐D‐glucamine artificial cerebrospinal fluid (NMDG ACSF) that included (in mM) 93 NMDG, 1.2 NaH_2_PO_4_, 2.5 KCl, 20 N‐2‐hydroxyethylpiperazine‐N‐2‐ethanesulfonic acid (HEPES), 30 NaHCO_3_, 5 Na‐ascorbate, 2 thiourea, 25 glucose, 0.5 CaCl_2_, 10 MgSO_4_, and 3 Na‐pyruvate (GSH). After that, the mice were decapitated, and the brain was removed and attached to the vibratome bedplate. The coronal slices with a thickness of 300 μm containing the LA were sectioned at 0.18 mm/s in ice‐cold NMDG ACSF (VT1200s, Leica). The slices were then incubated for 12 min in NMDG ACSF at 33°C before being transferred to HEPES ACSF containing (in mM) 92 NaCl, 2.5 KCl, 20 HEPES, 30 NaHCO_3_, 1.2 NaH_2_PO_4_, 25 glucose, 5 Na‐ascorbate, 3 Na‐pyruvate, 2 MgSO_4_, 2 CaCl_2_, 2 thiourea, and 3 GSH at 28°C for at least 1 h. Slices were transferred to a recording chamber (Warner Instruments, USA) and constantly perfused with carbogenated recording solution (129 mM NaCl, 3 mM KCl, 3 HEPES, 2.4 mM CaCl_2_, 20 mM NaHCO_3_, 10 mM glucose, 1.3 mM MgSO_4_, and 1.2 mM KH_2_PO_4_) at a rate of 3 mL/min at 32°C with the aid of an in‐line solution heater (TC‐344B, Warner Instruments). All ACSF was carefully adjusted with the pH at 7.3–7.4, the osmolarity at 300–310 mOsm/kg, and bubbled continuously with 95% O_2_/5% CO_2_.

An infrared‐sensitive CCD (charge‐coupled device) camera with a 40× water‐immersion lens (BX51WI, Olympus) was utilized to visualize the neurons for patch‐clamp recordings. Patch pipettes (5–7 MΩ) were filled with internal solution containing (in mM) 130 K‐gluconate, 5 KCl, 2 MgCl_2_, 10 HEPES, 0.6 EGTA, 0.3 Na‐GTP, and 2 Mg‐ATP (pH: 7.2–7.3, osmolarity: 285–290 mOsm/kg) and pulled from 1.5 mm borosilicate glass capillaries (VitalSense Scientific Instruments, China) on a horizontal micropipette puller (P1000, Sutter Instruments). To record the CNO‐evoked change in membrane potentials, the hM4Di‐expressing neurons were identified and approached for recording in the current clamp mode at a holding current of 0 pA. The signals were acquired with a Multiclamp 700B amplifier and an Axon 1550B digitizer, low‐pass filtered at 2.8 kHz, digitized at 10 kHz, and analyzed with Clampfit 11 software (Molecular Devices, USA). The data were acquired from the recordings with a series resistance of no more than 30 MΩ. If the series resistance changed by more than 20%, the recording was stopped immediately.

### 2.8 Immunohistochemistry and imaging

The immunohistochemistry and imaging were performed to visualize virally traced fluorescence signals and characterize the morphology of microglia. The mice were deeply anesthetized with pentobarbital sodium (20 mg/kg, i.p.) before following sequential transcardial perfusion of saline and 4% (w/v) paraformaldehyde (PFA). The brains were then carefully removed and post‐fixed in 4% PFA overnight at 4°C. Coronal slices (40 μm) were sectioned on a cryostat (Leica CM1860) and used for immunofluorescence after brain cryoprotection in 30% (w/v) sucrose. The collected slices were preserved at −20°C in a cryoprotectant solution containing PBS, 20% ethylene glycol (v/v), and 30% glycerol (v/v) for further staining or imaging. For immunofluorescence staining, the brain slices were washed three times with PBS and then blocked for 1.5 h at room temperature with 10% donkey serum dissolved in PBS with 5% Triton X‐100. The slices were then incubated with a specific primary antibody diluted in 3% donkey serum and Triton X‐100 for 24 h at 4°C. Next, slices were incubated for 1.5 h with appropriate fluorophore‐conjugated secondary antibody at room temperature. The primary antibodies include antibodies for Iba‐1 (1:500, Goat, AbCam), CD68 (1:500, Mouse, AbCam), MHCII (1:500, Mouse, AbCam), PSD95 (1:500, Rabbit, Cell signaling), and Gephyrin (1:500, Mouse, Synaptic systems), and the secondary antibodies include Alexa fluor 488‐anti‐Goat secondary antibody (1:500, Invitrogen), Alexa fluor 594‐anti‐Mouse secondary antibody, and Alexa fluor 594‐anti‐Rabbit secondary antibody (1:500, Invitrogen). The brain slices were mounted and coverslipped for imaging after being counterstained with DAPI (1:1000, No. D9542, Sigma‐Aldrich) for 2 min and three washes. The confocal microscopes (LSM880 and LSM980, ZEISS, Germany) were used to image and visualize the slices' fluorescence signals, and ImageJ (NIH, USA) was used to conduct the analysis.

### 2.9 Microglial quantification

Confocal images of brain slices with Iba‐1 immunofluorescence were captured using a Zeiss LSM 880 or Zeiss LSM 980 (40× oil immersion lens). To achieve three‐dimensional (3D) analysis of microglia, the images were morphologically analyzed as follows. “Bitplane” function built into Imaris 9.6.2 software was used to analyze 512 × 512‐pixel‐resolution images stacked in 1‐μm steps, and the number of branch points and process length were measured using the “Filaments” function. To analyze microglial engulfment, Imaris software was used to build a three‐dimensional surface rendering of the microglia, and a threshold was set to reconstruct microglial processes precisely. Puncta for PSD95^+^ or Gephyrin^+^ were reconstructed using the “Spots” algorithm built into the Imaris software. A plugin of “Split into Surface Objects” in the Imaris MATLAB (MathWorks) was used to count the puncta for PSD95^+^ or Gephyrin^+^ thoroughly in the membrane surface of Iba‐1‐positive microglia. The experimenter randomly selected two images containing at least ten microglia from individual animals for reconstruction.

### 2.10 Behavioral tests

In order to examine the possible behavioral effects of chronic moderate noise exposure in mice, we selected exploration‐based approach‐avoidance conflict tests that exploit the tendency of rodents to avoid rather than approach potential dangers.^53^, ^54^ Among this class of behavioral assays, we used open‐field test (OFT), elevated plus maze (EPM), and light–dark Box (LDB) as experimental paradigms, conducted in that respective order. After each test, mice were given at least 1 h of rest to recover.

#### 2.10.1 Open‐field test

The OFT apparatus (50 × 50 × 40 cm) included a 25 × 25 cm center square, and a wall‐surrounded peripheral region. At the start of the test, individual mouse was placed in a corner and allowed 5 min of free exploration, with their movements recorded by video camera. EthoVision XT software (Noldus, Netherlands) was used to compute time spent in the center area and total distance traveled, with less exploration time in center region considered anxiety‐like behavior.^55^ The OFT apparatus was thoroughly cleaned with 75% ethanol to remove all possible odor cues after each test.

#### 2.10.2 Elevated plus maze test

The EPM apparatus was a plus‐shaped maze with two closed arms (30 × 6 × 20 cm), two open arms (30 × 6 cm) perpendicular to the closed arms, and a center region (6× 6 cm), all raised approximately 100 cm above the floor. At the start of tests, individual mice were placed in the center facing a closed arm and allowed 5 min of free exploration, recorded by a video camera above the apparatus. EthoVision XT software (Noldus, Netherlands) was used to measure time spent in the open arms versus closed arms in each video, with less time in open arms considered representative of anxiety‐like behavior.^56^ The EPM box was thoroughly cleaned with 75% ethanol after each use to remove odors.

#### 2.10.3 Light–dark box test

The LDB test boxes included equal‐sized light and dark chambers (20 × 15 × 30 cm) separated by a wall with an open door (5 × 5 cm) to allow free exploration of either chambers. At the start, individual mice were placed in the light chamber, and their movements throughout the LDB apparatus were recorded by an overhead video camera for 15 min. The travel trajectories and time in either chambers in each video were also measured using EthoVision XT software (Noldus, Netherlands), with less time in the light zone considered representative of anxiety behavior.^57^ As with other test apparatuses, the LDB was thoroughly cleaned with 75% ethanol to eliminate odors that might influence other animals or replicate tests.

### 2.11 Statistical analysis

Statistical analyses and data visualization were conducted with GraphPad Prism 8.0.2 (Graph Pad Software, USA) and Origin 2023 (OriginLab Software, USA) software, and were used for statistical analysis and graph charting. Assumptions of normality in data distributions were checked by Shapiro–Wilk test. For normally distributed data sets, we applied two‐tailed unpaired Student's *t*‐tests to compare the mean of two independent groups, or two‐way analysis of variance (two‐way ANOVA) followed by the Bonferroni post‐hoc test to assess the significance of differences in the means of three or more independent groups. For data sets that do not meet assumptions of normal distribution, Mann–Whitney U‐tests were applied for comparisons of means between two groups. The Correlation Plot app in Origin software was used to generate Pearson's correlation coefficient matrices. All numerical data are expressed as means ± standard error of the mean (SEM). Significance levels are indicated as *p < 0.05, **p < 0.01, and ***p < 0.001.

## 3 RESULTS

### 3.1 Chronic moderate noise exposure elicits anxiety‐like behavior in mice

**FIGURE 1:**
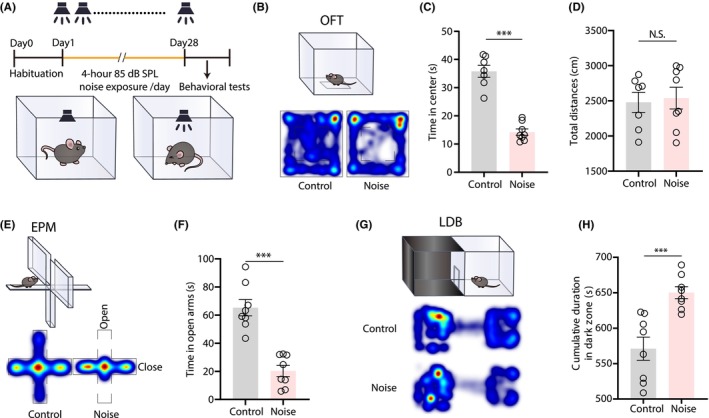
Chronic exposure to moderate noise induces anxiety‐like behavior in mice. (A) Timeline for noise exposure and behavioral tests (top). Schematic for noise exposure in a sound‐proof chamber (bottom). (B) Schematic (top) and representative heatmaps (bottom) for the open‐field test (OFT). (C and D) Summarized data for time spent in the center of OFT (C, *t*(13) = 9.381, *p* < 0.0001, *n* = 7 or 8 mice/group) and total distance traveled by noise‐exposed and control mice (D, *t*(13) = 0.2846, *p* = 0.7805, *n* = 7 or 8 mice/group). (E) Schematic (top) and representative heatmaps (bottom) for elevated plus maze (EPM). (F) Summarized data for time spent in the open arms of EPM (*t*(14) = 6.407, *p* < 0.0001, *n* = 8 mice/group). (G) Schematic (top) and representative heatmaps (bottom) for light–dark box (LDB). (H) Summarized data for time in dark zones of LDB (*t*(14) = 4.305, *p* = 0.0007, *n* = 8 mice/group). Data are expressed as means ± s.e.m. ***p < 0.001. NS, not significant. Student's unpaired *t*‐tests were used for (C), (D), (F), and (H). Details of the statistical analyses are presented in Table S1.

Mice were exposed to white noise (85‐dB SPL, 4 h/day for 28 days) in a sound‐attenuated chamber (Figure 1A). This chronic noise exposure reliably elicits anxiety‐like behaviors in the open‐field test (OFT), elevated plus maze (EPM), and light–dark box, as reported recently by our laboratory.^45^ In particular, in the OFT, noise‐exposed mice spent significantly less time in the open‐field center compared to control mice (Figure 1B,C), with no difference in total travel distance (Figure 1D). Similarly, noise‐exposed mice also spent less time in the open arms of the EPM (Figure 1E, F), indicating an anxiety‐like response to noise. Finally, in the light–dark box (LDB) test, noise‐exposed animals spent more time in the dark zone than control mice (Figure 1G,H). These behavioral tests thus consistently demonstrated that chronic non‐traumatic noise exposure could elicit anxiety‐like behaviors.

### 3.2 Noise‐induced anxiety involves the LA

**FIGURE 2:**
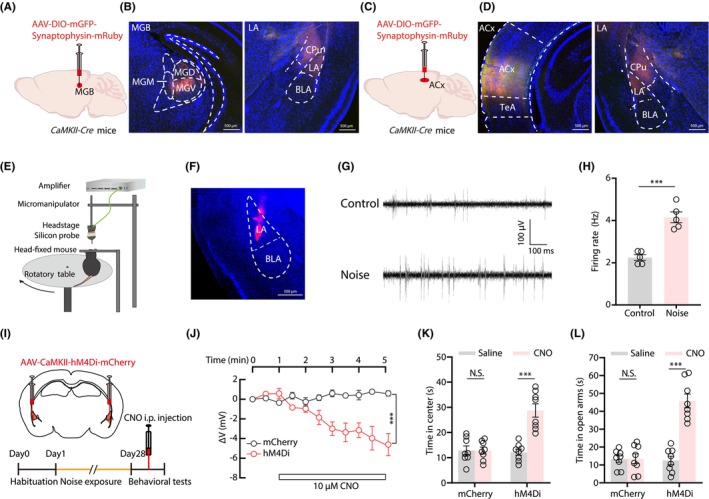
The LA is involved in chronic moderate noise exposure‐induced anxiety‐like behaviors. (A) Schematic for virus injection in the MGB of *CaMKII*‐Cre mice. (B) Example image of mRuby signals at the injection site in MGB (left) and MGB axon terminals in the LA (right). (C) Schematic for virus injection in the ACx of *CaMKII*‐Cre mice. (D) Example image of mRuby signals at the injection site in ACx (left) and ACx axon terminals in the LA (right). (E) Schematic of the setup for recording extracellular neuronal firing activity in head‐fixed mice using silicon probes. (F) Histological confirmation of the DiI‐labeled electrode track. (G and H) Representative voltage traces (G) and summarized average firing rates (H, *t*(8) = 6.587, *p* = 0.0002, *n =* 5 mice/group) of spontaneous action potentials recorded in the LA of noise‐exposed and control mice. (I) Schematic (top) and timeline (bottom) for chemogenetic inactivation of the LA. (J) Time course of CNO‐induced changes in membrane potentials of hM4Di‐expressing LA neurons (virus × time interaction, *F* (10, 55) = 5.931, *p* < 0.0001; main effect of virus, *F* (1, 55) = 63.57, *p* < 0.0001, *n* = 3 or 4 cells from 3 mice/group). (K) Time spent in the center of OFT in mice treated with saline or CNO (virus × drug interaction, *F* (1, 28) = 21.08, *p* < 0.0001; main effect of drug, *F* (1, 28) = 20.56, *p* < 0.0001, *n* = 8 mice/group). (L) Time spent in the open arms of EPM in mice treated with saline or CNO (virus × drug interaction, *F* (1, 28) = 34.95, *p* < 0.0001; main effect of drug, *F* (1, 28) = 35.73, *p* < 0.0001, *n* = 8 mice/group). Data are expressed as the means ± s.e.m. ***p < 0.001. NS, not significant. Student's unpaired *t*‐tests were used for (H). Two‐way ANOVA with Bonferroni post‐hoc analysis was used for (J–L). Details of the statistical analyses are presented in Table S1.

The auditory cortex and thalamus are important auditory brain regions with substantial efferent projections. In order to explore the connections between the auditory nucleus and emotion‐related regions, we injected an AAV‐DIO‐mGFP‐synaptophysin‐mRuby or AAV‐DIO‐mCherry anterograde virus into the auditory cortex, which is involved in perceiving sound and the auditory thalamus of the medial geniculate body, which acts as the gatekeeper, in the *CaMKII‐Cre* mice. This strategy enabled visualization of presynaptic axon fibers.^58^ Tracing data showed that the medial geniculate body and the auditory cortex (Figure 2A–D; Figure S1) both innervated the LA, zona incerta, and striatum. Since noise information can reach the LA via these two dense projections, we investigated changes in the LA neuronal activity following chronic noise exposure. In vivo extracellular recordings using silicon probes in awake head‐fixed mice revealed that spontaneous firing rates increased in the LA of noise‐exposed mice compared to control mice (Figure 2E–H). These results suggested that increased activity in the LA could account for the development of anxiety‐like behaviors. We further explored this possibility through chemogenetic inhibition of the bilateral LA. Patch‐clamp recordings from hM4Di‐expressing LA neurons in acute coronal brain slices revealed significant hyperpolarization of membrane potentials in response to bath administration of clozapine N‐oxide (CNO) (Figure 2I,J). As expected, chemogenetic inactivation of bilateral LA via CNO (i.p. 5 mg/kg) injection completely blocked chronic noise‐evoked anxiety‐like responses in OFT and EPM tests (Figure 2K,L). Together, these results showed that hyperactivity in the LA following noise exposure was closely related to the anxiety‐like behaviors in mice.

### 3.3 LA microglial activation correlates with noise‐evoked anxiety‐like behaviors

**FIGURE 3:**
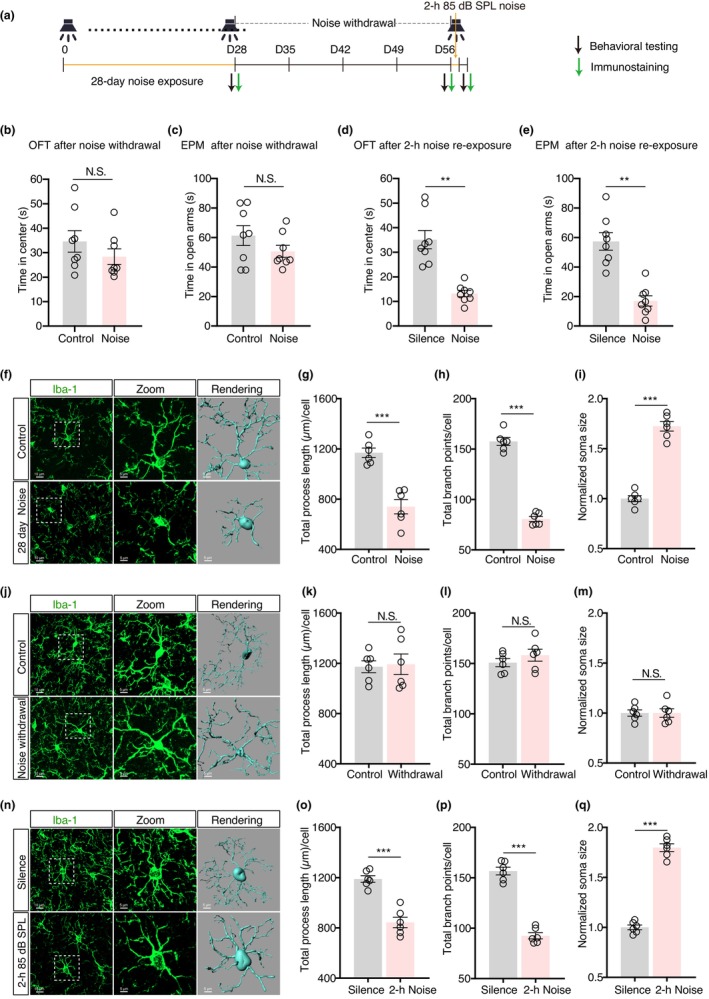
Microglial activation correlated with development of anxiety‐like behaviors. (A) Timeline of the experimental protocol. (B and C) Summarized data for time spent in the center of OFT (B, *t*(14) = 1.153, *p* = 0.2680, *n* = 8 mice/group) and time spent in the open arms of EPM (C, *t*(14) = 1.359, *p* = 0.1957, *n* = 8 mice/group) after 28 days of noise withdrawal following 28‐day treatment of moderate noise exposure. (D and E) Summarized data for time spent in the center of OFT (D, *t* (14) = 5.550, *p* < 0.0001, *n* = 8 mice/group) and time spent in the open arms of EPM (E, *t* (14) = 5.912, *p* < 0.0001, *n* = 8 mice/group) in noise‐withdrawal mice treated with acute noise. (F) Typical images of Iba‐1 immunofluorescence (left), magnified view of typical microglia (middle), and three‐dimensional (3D) reconstruction of microglia in the LA of 28‐day moderate noise group and control mice. (G–I) Summarized data of the total process lengths (G, *t*(10) = 6.300, *p* < 0.0001), total branch points (H, *t*(10) = 16.53, *p* < 0.0001), and normalized soma size (I, *t* (10) = 12.77, *p* < 0.0001) of Iba‐1^+^ microglia measured by the semi‐automated cell morphometry analysis tool in Imaris software (*n* = 6 mice/group). (J) Typical images of Iba‐1 immunofluorescence (left), magnified view of the typical microglia (middle), and 3D reconstruction of the LA microglia in the 28‐day noise withdrawal and control group. (K–M) Summarized data for total process lengths (K, *t* (10) = 0.2127, *p* = 0.8358), total branch points (L, *t*(10) = 1.032, *p* = 0.3262), and normalized soma size (M, *t*(10) = 0.0032, *p* > 0.9999) of Iba‐1^+^ microglia measured by the semi‐automated cell morphometry analysis tool in Imaris software (*n* = 6 mice/group). (N) Typical images of Iba‐1 immunofluorescence (left), magnified view of the typical microglia (middle), and 3D reconstruction of the LA microglia in the 2 h additional noise and silence control group. (O–Q) Summarized data of the total process lengths (O, *t* (10) = 7.025, *p* < 0.0001), total branch points (P, *t*(10) = 13.01, *p* < 0.0001), and normalized soma size (Q, *t* (10) = 17.61, *p* < 0.0001) of Iba‐1^+^ microglia measured by the semi‐automated cell morphometry analysis tool in Imaris software (*n* = 6 mice/group). Data are expressed as means ± s.e.m. **p < 0.01; ***p < 0.001. NS, not significant. Student's unpaired *t*‐tests were used for all figure panels. Details of the statistical analyses are presented in Table S1.

As prior chronic noise exposure can reportedly induce an acute stressor‐sensitive state in mice,^45^ we also found that mice treated with chronic sound exposure exhibited no anxiety‐like behaviors after 28 days of noise withdrawal following chronic sound exposure (Figure 3A–C). However, these noise‐treated mice indeed showed anxiety‐like symptoms upon exposure to a single 2‐h noise (85‐dB SPL) treatment after the 28‐day withdrawal compared to control animals (Figure 3D,E). Interestingly, we also observed concurrent changes in the morphology of LA microglia of noise‐exposed mice. Following noise exposure, microglia had decreased overall process length and number of branch points but increased soma size (Figure 3F–I), indicating microglial activation.^48^ The microglial morphology returned to normal (i.e., inactive state) after 28 days of noise withdrawal (Figure 3J–M). However, microglia underwent a similar activation pattern in response to the acute (2 h) noise exposure at 85‐dB SPL noise, which did not produce any obvious changes in the LA microglia of control animals (Figure 3N–Q). As a result, these findings demonstrated that the activation state of microglia coincided with the development of anxiety‐like behaviors in mice with chronic exposure to moderate noise.

### 3.4 Crucial role of LA microglial activation in noise‐induced anxiety

**FIGURE 4:**
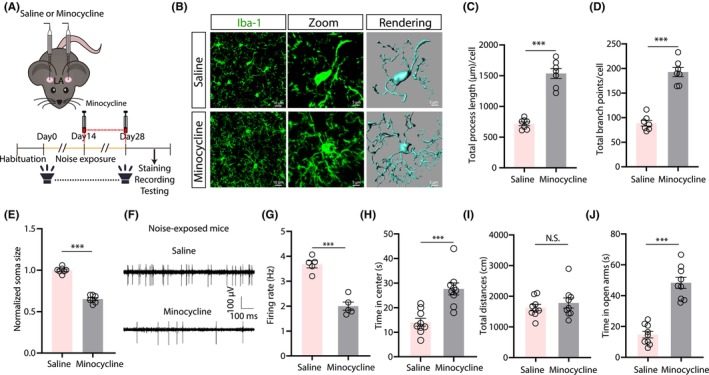
Microglial activation in the LA contributes to chronic moderate noise‐induced anxiety‐like behaviors. (A) Schematic for local minocycline injection in the LA to inactivate microglia, and the timeline for noise exposure, minocycline injection, staining, and behavioral tests. (B) Typical images of Iba‐1 immunofluorescence (left), magnified view of the typical microglia (middle), and 3D reconstruction of microglia in the LA of 28‐day moderate noise‐exposed mice treated with minocycline or saline. (C–E) Summarized data of and total process lengths (C, *t*(12) = 9.406, *p* < 0.0001, *n* = 7 mice/group), the total branch points (D, *t*(12) = 9.545, *p* < 0.0001, *n* = 7 mice/group), and normalized soma size (E, *t*(12) = 15.57, *p* < 0.0001, *n* = 7 mice/group) of Iba‐1^+^ microglia measured by the semi‐automated cell morphometry analysis tool in Imaris software. (F and G) Typical voltage traces (F) and summarized data of spontaneous firing rates in the LA of 28‐day moderate noise‐exposed mice injected with minocycline or saline (G, *t*(8) = 7.774, *p* < 0.0001, *n* = 5 mice/group). (H–I) Summarized data of time spent in the center (H, *t*(16) = 4.632, *p* = 0.0003, *n* = 9 mice/group) and total distance traveled (I, *t*(16) = 0.7691, *p* = 0.4530, *n* = 9 mice/group) in the OFT; time spent in the open arms of the EPM (J, *t*(16) = 8.039, *p* < 0.0001, *n* = 9 mice/group) in 28‐day moderate noise‐exposed mice treated with minocycline or saline. Data are expressed as the means ± s.e.m. ***p < 0.001. NS, not significant. Student's unpaired *t*‐tests were used for (C–E), (G–J). Details of the statistical analyses are presented in Table S1.

To investigate the role of LA microglial activation in noise‐evoked anxiety, the microglial suppressant, minocycline, was administered bilaterally at the LA to inhibit microglial activation starting from the 15th day after noise treatment. A lack of change in the microglial morphology in noise‐exposed animals treated with minocycline indicated that continuous minocycline treatment could efficiently inhibit microglial activation (Figure 4A–E). In vivo extracellular electrophysiology recordings also demonstrated that spontaneous firing rates of LA neurons significantly differed between noise‐exposed mice treated with minocycline and saline controls (Figure 4F,G). Thus, minocycline treatment prevented changes in microglial morphology and spontaneous firing of LA neurons in noise‐exposed mice. In accordance with these findings, minocycline treatment could also prevent the development of noise‐induced anxiety behaviors in OFT and EPM (Figure 4H–J). Taken together, these results revealed that microglial activation in the LA was crucial for the development of moderate noise‐evoked anxiety‐like behaviors.

### 3.5 Selective microglial phagocytosis of inhibitory synapses in the LA

**FIGURE 5:**
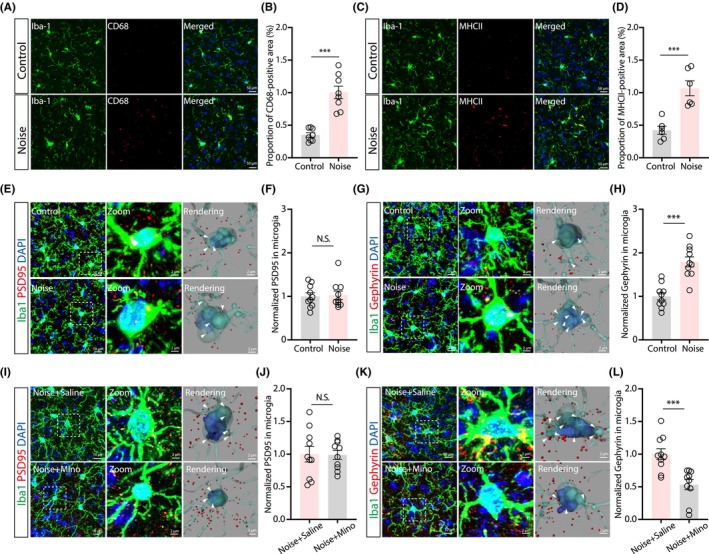
Microglia‐mediated phagocytosis of postsynaptic components. (A and B) Typical images of immunostaining for Iba‐1 and CD68 (A), and quantification of co‐localized immunofluorescence signal (B, *t*(14) = 66.413, *p* < 0.0001, *n* = 8 mice/group). (C and D) Typical images of immunostaining with antibodies against Iba‐1 and MHCII (C), and quantification of co‐localized signals (D, *t*(10) = 9.406, *p* = 0.0006, *n* = 6 mice/group). (E–H) Typical images of Iba‐1‐positive microglia containing PSD95 (E) or gephyrin (G) puncta in the LA of 28‐day moderate noise‐treated and control mice, and the summarized data (F, *U* = 48, *p* = 0.8973; H, *t*(18) = 5.562, *p* < 0.0001, *n* = 10 mice/group). Arrowheads indicate the inclusion of target protein puncta within the microglia. (I–L) Typical images of Iba‐1‐positive microglia containing PSD95 (I) or gephyrin (K) puncta in the LA of 28‐day moderate noise‐exposed mice treated with minocycline (Mino) or saline, and summarized data (J, *t*(18) = 0.08296, *p* = 0.9348; L, *U* = 8, *p* = 0.0006, *n* = 10 mice/group). Arrowheads indicate target protein puncta within the microglia. Data are expressed as the means ± s.e.m. ***p < 0.001. NS, not significant. Student's unpaired t‐tests were used for (B), (D), (H), and (J). Mann–Whitney *U*‐tests for (F) and (L). Details of the statistical analyses are presented in Table S1.

Microglia are known to alter neuronal circuits during organismal development and in various diseases. We found that the relative level of CD68 and MHCII (major histocompatibility complex class II) in Iba‐1 positive microglia was markedly higher in noise‐exposed mice (Figure 5A–D), indicating microglial activation and increased phagocytosis. Furthermore, Pearson's correlation tests demonstrated strong associations between microglial morphological features and anxiety‐related behavior variables (Figure S2). Microglia mediate cell‐type‐specific phagocytosis,^59^ resulting in an imbalance between excitatory and inhibitory transmission. Therefore, to test whether microglia selectively engulf postsynaptic elements in the LA, we performed double immunofluorescence labeling of ionized calcium‐binding adapter molecule 1 (Iba‐1) with either PSD95 (a marker for glutamatergic postsynaptic elements) or gephyrin (a marker for inhibitory postsynaptic elements). In the LA of control mice, Iba‐1‐positive microglia co‐localized with each of the two markers, but chronic noise treatment induced a more significant increase in gephyrin^+^/Iba‐1^+^, but not PSD95^+^/Iba‐1^+^ puncta, in 3D‐rendered images. These findings suggested increased microglial engulfment of inhibitory synaptic components, suggesting an imbalance in favor of excitatory activity (Figure 5E–H). Minocycline treatment could also abolish this selective phagocytosis (Figure 5I–L). Collectively, these results demonstrated that chronic noise exposure causes microglia‐mediated selective phagocytosis of inhibitory postsynaptic components.

## 4 DISCUSSION

In the current study, chronic moderate noise exposure (28 days, 4 h/day) could reliably induce anxiety‐like behaviors in mice, in a manner dependent on increased LA activity. Noise‐exposed mice were susceptible to anxiety induced by acute noise after 28 days of noise withdrawal. These behaviors occurred in parallel with the alterations in microglial morphology and neuronal excitability in the LA. Alternatively, minocycline treatment attenuated the noise‐induced changes in microglia and anxiety‐like behaviors. Immunofluorescence staining revealed that microglia‐mediated selective phagocytosis of inhibitory synapses can contribute to the imbalance between excitation and inhibition, resulting in an overexcited LA and subsequent anxiety‐like behaviors.

As an important emotional center, many studies have revealed that the amygdala plays an essential role in anxiety‐like behaviors through its complex neuronal pathways.^19^ The basolateral amygdala, including the lateral nucleus, as well as basal and accessory‐basal nuclei, has been shown to regulate stress‐related anxiety behaviors.^20^ The LA, as a sensory interface, receives both cortical and thalamic inputs.^29^ Our tracing experiments further verified that noise information reaches the amygdala via these two routes. Optical activation of LA neurons has been observed to cause anxiety‐like behaviors, and artificially activating synaptic inputs to the BLA, such as the ventral tegmental region,^60^ can also reportedly cause anxiety‐like behaviors. Consistent with our recent findings, long‐term moderate noise stimulation similarly altered BLA neuronal activity.^45^ Consistent with this effect, BLA neurons showed enhanced excitability in mice with chronic stress.^31^

As brain‐resident immune cells, microglia are responsible for continuous surveillance of the parenchyma and can alter neural circuits via secreted inflammatory factors or phagocytosis.^36^, ^61^ Furthermore, microglia dynamically prune synapses, consequently influencing behaviors.^36^ During development, steroid‐induced microglial phagocytosis of astrocytes in the amygdala reportedly alters sexual differentiation of social circuits and related behaviors.^62^ In adults, microglia have been shown to participate in processes of forgetting,^63^ anxiety,^38^ and depression‐like behaviors,^48^ and microglial dysfunction has been implicated in chronic stress‐induced depression. Microglia‐mediated mPFC neuronal remodeling contributes to synaptic deficits and mood disorders via neuronal colony‐stimulating factor 1 in mice with chronic unpredictable stress.^64^ Moreover, activated microglia in the mPFC of mice with social defeat stress have been shown to promote neuronal atrophy, impair response attenuation, and cause social avoidance via TLR2/4‐dependent IL‐1α and tumor necrosis factor alpha.^65^ Alternatively, microglia‐mediated changes in BLA circuits are also closely related to neuropsychiatric disorders. For example, LPS treatment can activate microglia, which subsequently produce pro‐inflammatory cytokines in the BLA, causing anxiety and depression.^37^ Optogenetic stimulation of microglia expressing Hoxb8 in the BLA also results in increased anxiety.^38^ In addition, reactive microglia in the BLA are known to contribute to synaptic dysfunction and depression‐like behaviors in pain model mice.^40^ Furthermore, evidence in male rats suggests that inhibiting microglia in the amygdala can reverse stress‐induced abdominal pain.^41^

Microglia are also engaged in experience‐dependent neural circuit remodeling.^66^, ^67^ In the somatosensory system, whisker lesions cause microglia‐mediated loss of synapses in the barrel cortex via CX3CL1 and CX3CR1.^67^ In the visual system, microglia have been shown to engulf retinogeniculate synapses via CR3/C3 signaling.^68^ In addition, light deprivation can alter microglial motility and reduce their preference for synapses in the visual cortex.^66^ These studies show that sensory input‐responsive microglia can modify neuronal circuits in a context‐dependent manner,^66^ which also applies to the auditory system wherein acoustic trauma can activate microglia in both auditory and limbic brain regions.^33^ Loud noise‐induced hearing loss also reportedly increases activity in the auditory cortex via microglia.^5^ Sound‐induced auditory cortical and hippocampal gamma waves can activate microglia to engulf plaques in a mouse model of Alzheimer's disease.^42^ In addition, salicylate‐induced tinnitus‐like mice exhibit significant microglial activation in the auditory cortex and medial geniculate body.^43^ It is therefore reasonable that chronic noise exposure, as a sensory stimulus, can also induce changes in microglial morphology and function in brain regions responsive to acoustic stimuli.

Microglia act as scavengers that can produce neuronal overexcitation in epilepsy.^59^, ^69^ Consistent with this activity, our findings suggest that noise‐activated LA microglia selectively engulf neuronal components. Greater gephyrin engulfment compared to PSD95 engulfment by microglia suggests that microglia may preferentially engulf more inhibitory than excitatory postsynaptic components. Microglia expressing the GABAb1 receptor have also been shown to prune inhibitory synapses via a complement‐dependent mechanism in which GABA acts as a chemotactic signal to recruit microglia responsive to GABA‐b receptor, which subsequently degrades these inhibitory synapses.^70^ In addition, progranulin^−/−^ microglia were found to preferentially eliminate inhibitory synapses in the ventral thalamus.^71^ Activated microglia can also displace inhibitory presynaptic terminals from cortical neurons to promote synchronization of cortical neurons in the g‐frequency band.^72^ Visual deprivation increases whisker stimulation‐evoked responses in the visual cortex by microglia‐mediated removal of inhibitory synapses.^73^ However, the mechanism underlying microglia‐mediated selective phagocytosis remains unclear and merits further investigation. The principal neurons in the LA are inhibited by local GABA interneurons^74^ or inhibitory projections from the intercalated amygdala,^75^ or even the auditory cortex.^76^ However, whether microglia display pathway‐specific engulfment of inhibitory synapses requires cell‐type‐specific fluorescent labeling.

**FIGURE 6:**
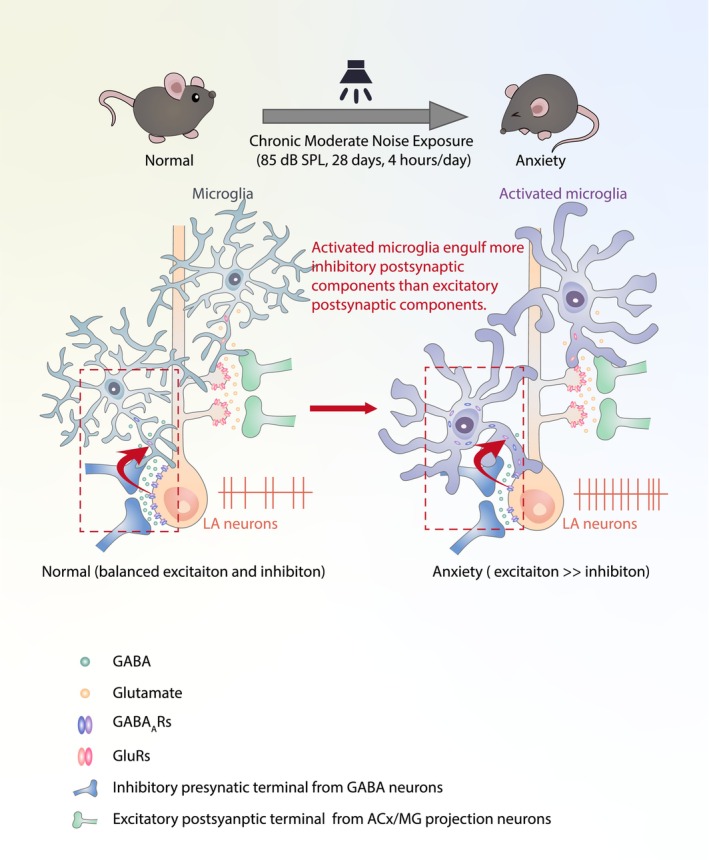
Proposed model of microglia‐mediated synaptic remodeling in the LA in the development of chronic moderate noise‐evoked anxiety behaviors. Chronic noise exposure activates LA microglia, which engulf more inhibitory than excitatory postsynaptic components, resulting in imbalanced excitatory transmission, potentially accounting for increased neuronal activity in the LA, and consequently leading to the development of anxiety‐like behaviors.

Anxiety is often followed by hearing disabilities, such as hearing loss or tinnitus, and hearing loss‐related anxiety was examined in another recent study.^7^ Acoustic trauma has been shown to affect limbic systems, including the cingulate cortex^77^ and hippocampus,^7^ and also enhances connectivity between the auditory system, amygdala, and hippocampus,^78^, ^79^, ^80^ resulting in anxiety‐like behaviors. Exposure to loud noise is widely used to experimentally induce hearing loss in laboratory animals.^7^, ^81^ However, non‐traumatic noise, such as noise at 85‐dB SPL in the present study, is frequently encountered in traffic, workplaces, and entertainment.^2^, ^3^, ^4^ Even long‐term exposure to ostensibly benign, moderate noise levels can impede hearing^13^ and negatively impact hippocampus‐dependent learning.^15^ Changes in neurotransmitter levels^82^ and synaptic transmission^45^ in the amygdala related to this type of noise exposure have also been observed and also contribute to development of anxiety‐like behaviors. In our study, we specifically set the noise at a non‐traumatic level, distinctly lower than traumatic noise levels examined in other previous studies. Our objective was to investigate the origins of neuronal hyperactivity, especially the role of microglial activity, diverging from the conventional focus on synaptic plasticity or neural circuitry found in the current literature.^7^, ^45^ Salicylate, a drug known to induce tinnitus, not only affects thalamocortical information transfer^83^ but can also elicit anxiety‐like behaviors.^84^, ^85^, ^86^ Salicylate enhances neural activity and induces changes in synaptic ultrastructure in the hippocampus.^85^, ^87^ Notably, chronic moderate noise exposure affects the amygdala, whereas acoustic trauma or salicylate exposure appears to influence hippocampal function. The observation that projections from the amygdala to hippocampus can play a role in anxiety‐like behaviors may reconcile this disparity.^26^ Imaging data, physiological recordings, and c‐Fos staining experiments in rodents collectively indicate that salicylate‐induced hyperactivity also occurs in the amygdala.^88^, ^89^, ^90^ Furthermore, salicylate has been shown to activate microglia,^43^ which could potentially occur in amygdala microglia, aligning well with our hypothesis that amygdala microglia can promote anxiety‐like behaviors. Under noise exposure, sound is channeled to the LA via auditory cortical and thalamic inputs, accompanied by heightened LA activity, as observed through in vivo electrophysiology recordings. The LA is positioned to bridge the auditory system and emotion center, and a maladaptive LA may consequently promote anxiety‐like behavior.^45^ Our observations that microglial activation in the LA can be blocked through chemogenetic inhibition in mice with chronic noise‐induced anxiety‐like behavior are consistent with previously reported contribution of microglia to overexcitation of the LA in stress‐induced anxiety.^37^ We therefore propose a model to explain the role of microglia in chronic noise‐induced anxiety in which microglia engulf more inhibitory postsynaptic components than excitatory components, shifting the balance toward excitation. This imbalance could potentially account for the elevated activity we observed in LA and subsequent development of anxiety‐like behaviors in mice (Figure 6).

Interestingly, mice with chronic noise‐induced anxiety become susceptible to stress, an effect closely associated with altered neuronal activity and microglial activation in the LA. However, the detailed neural basis for this priming effect requires extensive investigation in future work. Our finding that noise‐exposed mice exhibit enhanced susceptibility to acute stressors aligns well with microglia‐mediated two‐hit hypothesis,^91^, ^92^ in which an early stressor (the “first hit” of chronic moderate noise exposure) sensitizes microglial cells, resulting in an exaggerated increase in microglial activity upon exposure to subsequent stressor events (the “second hit” of acute noise exposure). This first insult thus induces a primed state in microglia, leading to faster and stronger activation in response to the second insult, and ultimately, an overactivated state. Although our findings suggest that selective synapse pruning contributes to increased neuronal activity in BLA, neuro‐inflammation due to microglia‐secreted factors cannot be ruled out.

The increasing noise pollution that accompanies societal modernization can severely negatively affect quality of life.^1^, ^16^ Considering the hazards posed by non‐traumatic noise that have been previously considered safe, public health policy should endeavor to minimize unnecessary noise exposure. It should also be noted that the neural mechanisms underlying noise‐induced anxiety are likely more complicated in humans than in mice. However, the present investigation highlights the potential long‐term impacts of noise pollution on health and suggests that LA microglia might serve as potentially effective targets in the development of treatments for anxiety.

## AUTHOR CONTRIBUTIONS

X. P., Y. M., and Y. L designed the studies, conducted most of the experiments and data analysis, and wrote the methods and materials of the manuscript. T.Y., Q. D, B. L, Y. L, R.G conducted some behavioral experiments. W.Z. and L.C. contributed to discussion. Z. Z., G.S., and H.W. were involved in the project's overall design, individual experiments, data analysis, supervision, and the writing of the manuscript.

## CONFLICT OF INTEREST STATEMENT

The authors declare no competing financial interests.

## Supporting information


Table S1.

Figure S1.
Figure S2.

## Data Availability

The datasets used and/or analyzed during the current study are available from the corresponding author on reasonable request.
