# Supplementary material for: Microglial activation in the lateral amygdala promotes anxiety‐like behaviors in mice with chronic moderate noise exposure
Source: CNS Neurosci Ther. 2024 Mar 11;30(3):e14674. doi: 10.1111/cns.14674 (PMC10927919; doi:10.1111/cns.14674)
Supplement: Supplementary file 1 — Table S1. [file CNS-30-e14674-s001.pdf]

Figure Panel	n/group	P value for Normality test	Primary statistic	*Post-hoc* test	Comparison	p value	F/t statistic
Fig. 1C	Control, n = 7 mice	0.63	two-tailed unpaired Student's t-test		Control vs. Noise	<0.0001	*t* (13) = 9.381
	Noise, n = 8 mice	0.25					
Fig. 1D	Control, n = 7 mice	0.24	two-tailed unpaired Student's t-test		Control vs. Noise	0.7805	*t* (13) = 0.2846
	Noise, n = 8 mice	0.19					
Fig. 1F	Control, n = 8 mice	0.61	two-tailed unpaired Student's t-test		Control vs. Noise	<0.0001	*t* (14) = 6.407
	Noise, n = 8 mice	0.11					
Fig. 1H	Control, n = 8 mice	0.22	two-tailed unpaired Student's t-test		Control vs. Noise	0.0007	*t* (14) = 4.305
	Noise, n = 8 mice	0.85					
Fig. 2H	Control, n = 5 mice	0.14	two-tailed unpaired Student's t-test		Control vs. Noise	0.0002	*t* (8) = 6.587
	Noise, n = 5 mice	0.64					
Fig. 2J	mCherry, n = 3 mice	All p value > 0.05	Two-way RM ANOVA	Bonferroni's multiple comparison	Group × time interaction	< 0.0001	F(10,55) = 5.931
	hM4Di-mCherry, n = 4 mice	All p value > 0.05					
Fig. 2K	mCherry Saline, n = 8 mice	0.53	Two-way RM ANOVA	Bonferroni's multiple comparison	Group × time interaction	< 0.0001	F(1,28) = 21.08
	mCherry CNO, n = 8 mice	0.72			mCherry Saline vs. CNO	> 0.9999	
	hM4Di-mCherry Saline, n = 8 mice	0.96			hM4Di-mCherry Saline vs. CNO	<0.0001	
	hM4Di-mCherry CNO, n = 8 mice	0.18					
Fig. 2L	mCherry Saline, n = 8 mice	0.35	Two-way RM ANOVA	Bonferroni's multiple comparison	Group × time interaction	< 0.0001	F(1,28) = 34.95
	mCherry CNO, n = 8 mice	0.35			mCherry Saline vs. CNO	> 0.9999	
	hM4Di-mCherry Saline, n = 8 mice	0.78			hM4Di-mCherry Saline vs. CNO	<0.0001	
	hM4Di-mCherry CNO, n = 8 mice	0.29					
Fig. 3B	Control, n = 8 mice	0.32	two-tailed unpaired Student's t-test		Control vs. Noise	0.268	*t* (14) = 1.153
	Noise, n = 8 mice	0.05					
Fig. 3C	Control, n = 8 mice	0.22	two-tailed unpaired Student's t-test		Control vs. Noise	0.1957	*t* (14) = 1.359
	Noise, n = 8 mice	0.12					
Fig. 3D	Control, n = 8 mice	0.11	two-tailed unpaired Student's t-test		Control vs. Noise	<0.0001	*t* (14) = 5.550
	Noise, n = 8 mice	0.97					
Fig. 3E	Control, n = 8 mice	0.89	two-tailed unpaired Student's t-test		Control vs. Noise	<0.0001	*t* (14) = 5.912
	Noise, n = 8 mice	0.77					
Fig. 3G	Control, n = 6 mice	0.69	two-tailed unpaired Student's t-test		Control vs. Noise	<0.0001	*t* (10) = 6.300
	Noise, n = 6 mice	0.33					
Fig. 3H	Control, n = 6 mice	0.58	two-tailed unpaired Student's t-test		Control vs. Noise	<0.0001	*t* (10) = 16.53
	Noise, n = 6 mice	0.09					
Fig. 3I	Control, n = 6 mice	0.87	two-tailed unpaired Student's t-test		Control vs. Noise	<0.0001	*t* (10) = 12.77
	Noise, n = 6 mice	0.87					
Fig. 3K	Control, n = 6 mice	0.75	two-tailed unpaired Student's t-test		Control vs. Withdrawal	0.8358	*t* (10) = 0.2127
	Withdrawal, n = 6 mice	0.22					
Fig. 3L	Control, n = 6 mice	0.47	two-tailed unpaired Student's t-test		Control vs. Withdrawal	0.3262	*t* (10) = 1.032
	Withdrawal, n = 6 mice	0.75					
Fig. 3M	Control, n = 6 mice	> 0.9999	two-tailed unpaired Student's t-test		Control vs. Withdrawal	> 0.9999	*t* (10) = 0.003155
	Withdrawal, n = 6 mice	0.53					
Fig. 3O	Silence, n = 6 mice	0.80	two-tailed unpaired Student's t-test		Silence vs. 2-h Noise	<0.0001	*t* (10) = 7.025
	2-h Noise, n = 6 mice	0.81					
Fig. 3P	Silence, n = 6 mice	0.53	two-tailed unpaired Student's t-test		Silence vs. 2-h Noise	<0.0001	*t* (10) = 13.01
	2-h Noise, n = 6 mice	0.22					
Fig. 3Q	Silence, n = 6 mice	0.96	two-tailed unpaired Student's t-test		Silence vs. 2-h Noise	<0.0001	*t* (10) = 17.61
	2-h Noise, n = 6 mice	0.92					
Fig. 4C	Saline, n = 7 mice	0.44	two-tailed unpaired Student's t-test		Saline vs. Minocycline	<0.0001	*t* (12) = 9.406
	Minocycline, n = 7 mice	0.76					
Fig. 4D	Saline, n = 7 mice	0.54	two-tailed unpaired Student's t-test		Saline vs. Minocycline	<0.0001	*t* (12) = 9.545
	Minocycline, n = 7 mice	0.66					
Fig. 4E	Saline, n = 7 mice	0.71	two-tailed unpaired Student's t-test		Saline vs. Minocycline	<0.0001	*t* (12) = 15.57
	Minocycline, n = 7 mice	0.39					
Fig. 4G	Saline, n = 5 mice	0.78	two-tailed unpaired Student's t-test		Saline vs. Minocycline	<0.0001	*t* (8) = 7.774
	Minocycline, n = 5 mice	0.41					
Fig. 4H	Saline, n = 9 mice	0.64	two-tailed unpaired Student's t-test		Saline vs. Minocycline	0.0003	*t* (16) = 4.632
	Minocycline, n = 9 mice	0.15					
Fig. 4I	Saline, n = 9 mice	0.63	two-tailed unpaired Student's t-test		Saline vs. Minocycline	0.453	*t* (16) = 0.7691
	Minocycline, n = 9 mice	0.14					
Fig. 4J	Saline, n = 9 mice	0.58	two-tailed unpaired Student's t-test		Saline vs. Minocycline	<0.0001	*t* (16) = 8.039
	Minocycline, n = 9 mice	0.62					
Fig. 5B	Control, n = 8 mice	0.14	two-tailed unpaired Student's t-test		Control vs. Noise	<0.0001	*t* (14) = 6.413
	Noise, n = 8 mice	0.67					
Fig. 5D	Control, n = 6 mice	0.78	two-tailed unpaired Student's t-test		Control vs. Noise	0.0006	*t* (10) = 4.947
	Noise, n = 6 mice	0.10					
Fig. 5F	Control, n = 10 mice	0.75	Mann-Whitney U test		Control vs. Noise	0.8973	*U* = 48
	Noise, n = 10 mice	0.01					
Fig. 5H	Control, n = 10 mice	0.74	two-tailed unpaired Student's t-test		Control vs. Noise	<0.0001	*t* (18) = 5.562
	Noise, n = 10 mice	0.98					
Fig. 5J	Noise+Saline, n = 10 mice	0.61	two-tailed unpaired Student's t-test		Noise+Saline vs. Noise+Mino	0.9348	*t* (18) = 0.08296
	Noise+Mino, n = 10 mice	0.45					
Fig. 5L	Noise+Saline, n = 10 mice	0.65	Mann-Whitney U test		Noise+Saline vs. Noise+Mino	0.0006	*U* = 8
	Noise+Mino, n = 10 mice	0.03					
