# Supplementary material for: Microglial activation in the lateral amygdala promotes anxiety‐like behaviors in mice with chronic moderate noise exposure
Source: CNS Neurosci Ther. 2024 Mar 11;30(3):e14674. doi: 10.1111/cns.14674 (PMC10927919; doi:10.1111/cns.14674)
Supplement: Supplementary file 2 — Figure S1. Figure S2. [file CNS-30-e14674-s002.docx]

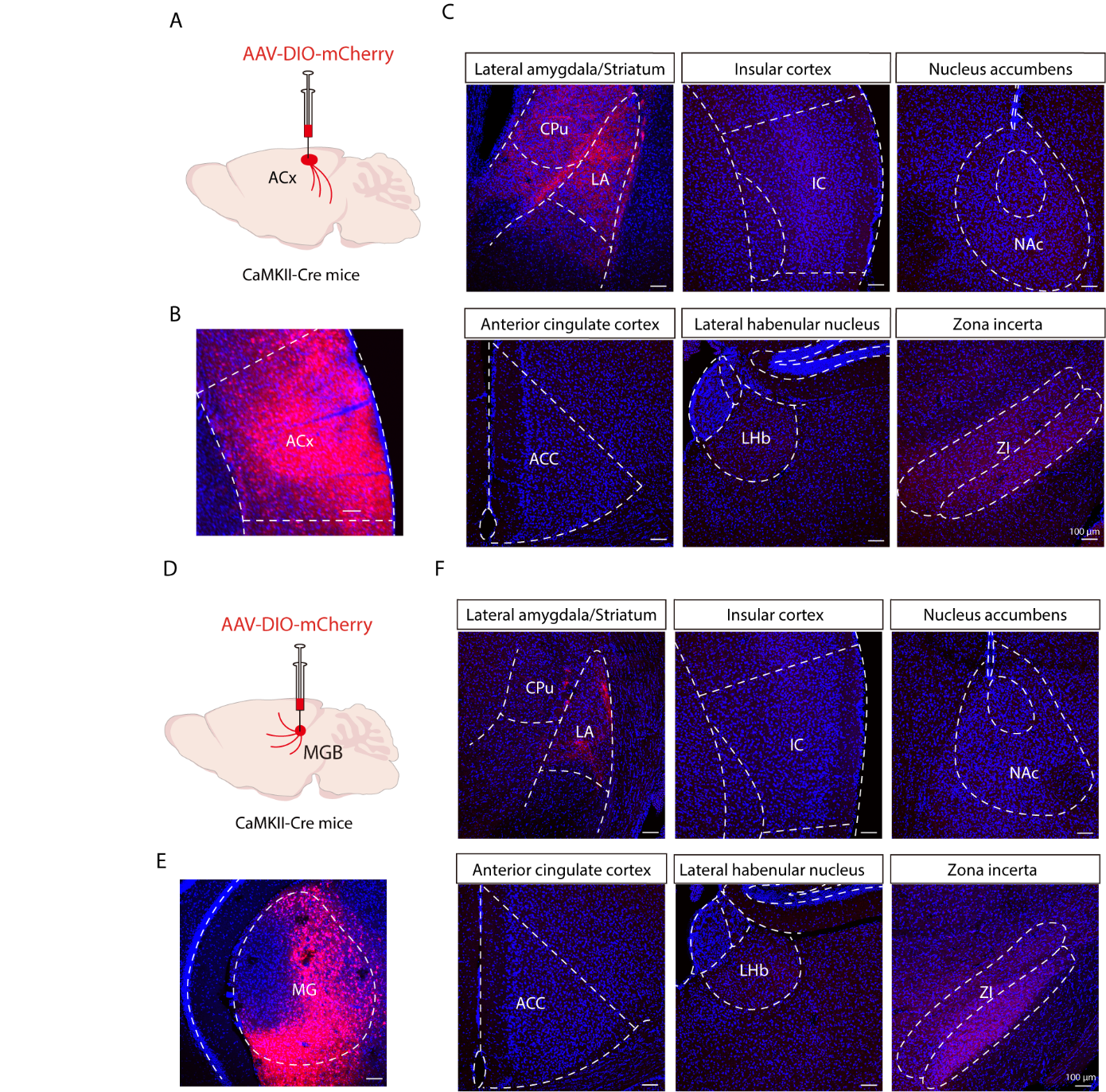


**Figure S1. Anterograde tracing of connections between the auditory system and limbic brain regions.** (**A**) Schematic for viral injection in the ACx of *CaMKII-Cre* mice. (**B**) A representative image of viral expression in the ACx of *CaMKII-Cre* mice. Scale bar, 100 μm. (**C**) Representative images of immunofluorescence signals in the indicated regions. (**D**) Schematic for viral injection in the MG of *CaMKII-Cre* mice. (**E**) A representative image of viral expression in the MG of *CaMKII-Cre* mice. Scale bar, 100 μm. (**F**) Representative images of fluorescence signals in the indicated regions. Note: ACC, anterior cingulate cortex; ACx, auditory cortex; CPu, caudate putamen; ICx, insular cortex; LA, lateral amygdala; LHb, lateral habenular nucleus, MG, medial geniculate body; NAc, nucleus accumbens; ZI, Zona incerta.


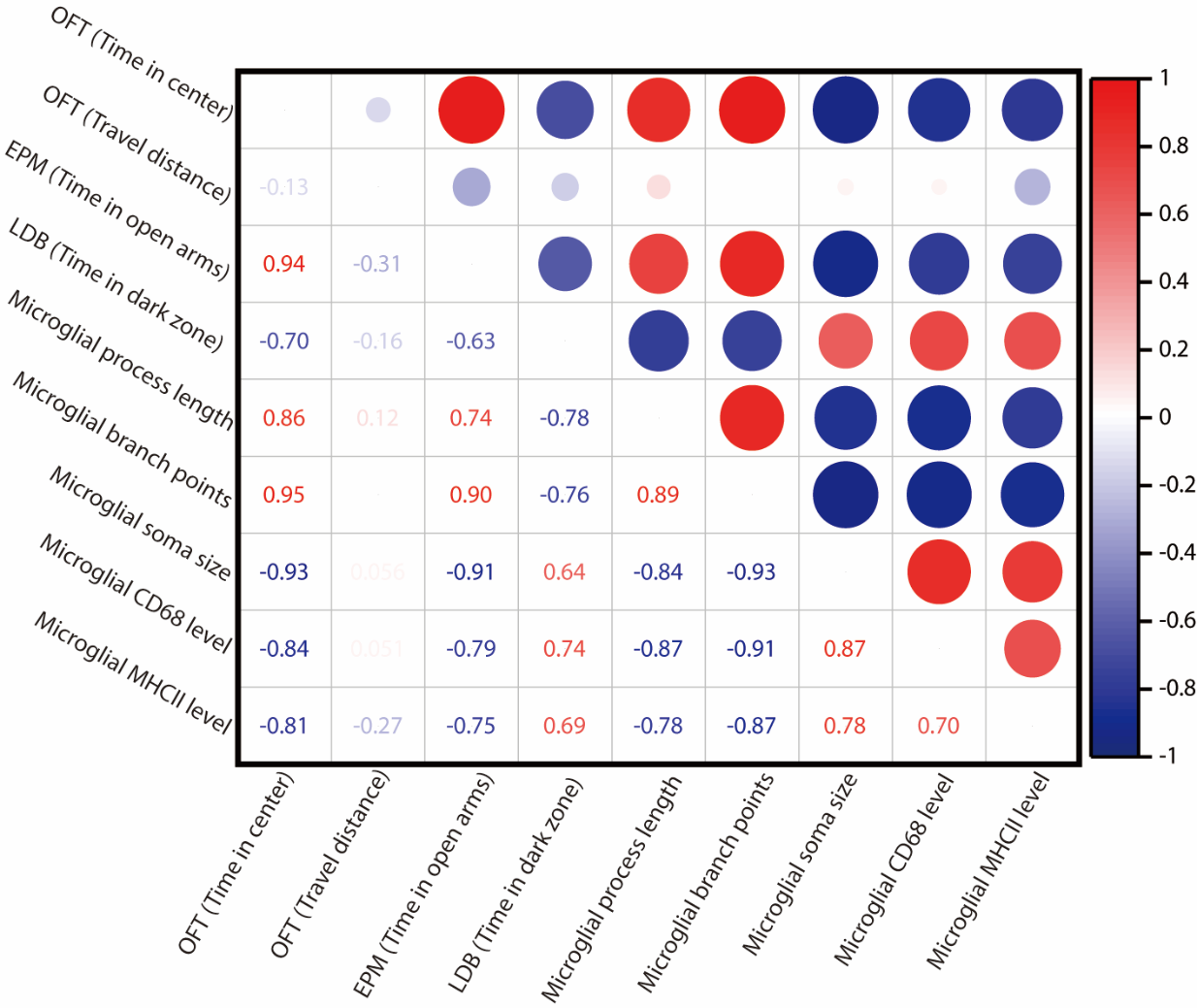


**Figure S2. Correlation analysis of potential associations between variables.** A symmetric correlation matrix created using the "Correlation Plot" plugin in Origin software. The colours and numbers represent the degree of pairwise correlation based on Pearson’s coefficient. Self-self correlations are blank. Significant correlations are colored in either red (positive) or blue (negative) hues.
